# A thermodynamic basis for teleological causality

**DOI:** 10.1098/rsta.2022.0282

**Published:** 2023-08-07

**Authors:** Terrence W. Deacon, Miguel García-Valdecasas

**Affiliations:** ^1^ Department of Anthropology, University of California, Berkeley, CA, USA; ^2^ Department of Philosophy, Universidad de Navarra, Pamplona, Navarra, Spain

**Keywords:** teleodynamics, autogen, self-organization, entropy production, evolution

## Abstract

We show how distinct terminally disposed self-organizing processes can be linked together so that they collectively suppress each other's self-undermining tendency despite also potentiating it to occur in a restricted way. In this way, each process produces the supportive and limiting boundary conditions for the other. The production of boundary conditions requires dynamical processes that decrease local entropy and increase local constraints. Only the far-from-equilibrium dissipative dynamics of self-organized processes produce these effects. When two such complementary self-organizing processes are linked by a shared substrate—the waste product of one that is the necessary ingredient for the other—the co-dependent structure that results develops toward a self-sustaining target state that avoids the termination of the whole, and any of its component processes. The result is a perfectly naturalized model of teleological causation that both escapes the threat of backward influences and does not reduce teleology to selection, chemistry or chance.

This article is part of the theme issue ‘Thermodynamics 2.0: Bridging the natural and social sciences (Part 1)’.

## 1. Introduction

Teleology is one of the most notoriously difficult concepts in the history of science and philosophy. Early enlightenment thinkers like Francis Bacon (1561–1626) or Spinoza (1632–1677) saw it as a misunderstanding of physical causality that obfuscated the logic of scientific explanation. In Bacon's perspective, ‘final causes […] lead to difficulties in science and tempt us to amalgamate theological and teleological points of doctrine’ [1]. Spinoza argued that ‘this doctrine of final causes turns Nature completely upside down, for it regards as an effect that which is in fact a cause, and vice versa’ [2]. This conceptual inversion of the cause-effect relation, sometimes called backward causation, has done much to discredit teleology. It implied that an unrealized state is able to influence a current physical process to bring that state into existence. This is of course physically implausible. The reality is somewhat less exotic. Human agents experience purposeful action as a disposition to achieve an end that is represented in advance as a possible form which somehow influences the selection of efficient means to achieve it. Of course, human agency evolved from the less-developed but nonetheless end-directed dynamics that is characteristic of all life forms. So, the minimal basis of teleological causality should be reflected in the organization of even the simplest living systems. This suggests that we should be able to identify a primitive homologue of this pre-specified form and determine how it is able to organize work to increase the probability that its form is realized.

Twentieth-century theorists endeavoured to overcome the problematic implications of teleological cause by reducing it to a mechanistic process, such as cybernetic feedback regulation (e.g. [3]), treating it as a programme or algorithm (e.g. [4]), replacing it with a merely descriptive concept like ‘teleonomy’ [5] or attributing its apparent nature to Darwinian selection, as in so-called ‘selected effect theories’ [6–8]. Rosenbluth's mechanisms, Pittendrigh's teleonomy and selection processes will be briefly reviewed below. In each case, teleological causality is treated as an epiphenomenon whose misleading appearance is due to incomplete analysis. So, although teleology is treated as a given in the social and anthropological sciences, it has become almost an unspoken rule that teleological explanation should play no explanatory role in the natural sciences. This includes biology, where the apparently teleological concepts of function and adaptation seem indispensable to account for the metabolic and molecular processes that make a trait functional or adaptive.

Explaining the operations of the simplest living processes requires straddling the distinction between mechanistic and teleological accounts. Even framed in thermodynamic terms, the teleological framing seems unavoidable. Organisms are described as performing chemical work in order to maintain themselves far-from-equilibrium, at the expense of ‘exporting’ entropy into their surroundings at a rate faster than they reduce it internally. To accomplish this, they must somehow use the second law against itself. So, a thermodynamic account of this most minimal form of teleological causality must make sense of this curious twist of thermodynamic logic by which the effects of the second law are temporarily locally reversed.

In this article, we provide a conceptual reanalysis of teleological processes in biology that both retain their future-oriented end-directed character and yet are entirely consistent with thermodynamic principles. We do this in seven sections.

Section 1 briefly reviews the challenges that the concept of teleology has posed for thermodynamic theories over the years. Section 2 summarizes how teleological issues in biology were sidestepped by appealing to natural selection theory. Section 3 provides an intuitive characterization of teleological phenomena and spells out some of the implications that end-directed processes have in thermodynamic contexts. Section 4 introduces the critical distinction between terminal and target-directed processes. Terminal processes develop spontaneously toward some maximum state beyond which further change is no longer possible driven by external conditions. Target-directed processes develop toward end states that are not terminal maxima but reflect local system-internal stable values. Section 5 discusses how far-from-equilibrium self-organizing processes develop toward terminal states that are locally far-from-equilibrium, but intrinsically unstable and dependent on extrinsic conditions. Section 6 explains how reciprocally linked interdependent self-organizing processes can compensate for each other's tendency to terminate. This coupling creates a disposition to project the form of this relation into the future. Section 7, the conclusion, summarizes how this distinctive configuration of thermodynamic processes can meet the challenges identified by Bacon and Spinoza and provide an explanation of natural teleological processes free of explanatory conundrums.

## 2. Teleology versus thermodynamics

Since the enlightenment, the concept of teleology has been haunted by the spectre of backward causality. With the development of thermodynamics in the middle of the nineteenth century, the plausibility of backward causality encountered a more subtle and sophisticated objection than the early philosophers of the Enlightenment could have imagined. The second law of thermodynamics introduced a ubiquitous time-asymmetry into mechanistic theories that Newtonian mechanics lacked. In simple terms, it required that more organized states of matter spontaneously and inevitably tend to degrade toward less organized states, and that the reverse is astronomically unlikely. The glaring exception is the end-directed dynamics that are characteristic of living and mental processes which apparently run counter to this ubiquitous trend.

In one of the more enigmatic contributions to the new field of thermodynamics, Maxwell [9] described a thought experiment that implicitly contrasted the physics of the second law with mental causality. This is the problem of Maxwell's demon. In this familiar conundrum, Maxwell suggested that if it were possible for a microscopic being to assess the relative velocity of molecules in two containers linked by a channel, and then was able to control which specific molecules passed from one container to the other, it would be possible to reverse the second law and progressively reduce the entropy of the whole system. Of course, to do this the demon would need to ensure that only molecules with relatively higher velocities would flow in one direction through the channel and that those with relatively lower velocities would flow in the reverse direction. As a result, one container would tend to heat up and the other would cool, which would also increase the potential for the system to do work, indefinitely. This outcome seemed obviously absurd, but explaining exactly why was not so clear.

Though fanciful and paradoxical, this thought experiment highlighted that in some way the end-directed actions of the demon constituted a disposition inverse to the second law. The implications of this relationship between purposive phenomena and thermodynamics were insightfully summarized by David Watson in a 1930 [10] *Science* article when he said: ‘Thought interferes with the probability of events, and, in the long run, therefore, with entropy’. But how?

Over the course of the twentieth century, various theoretical arguments have endeavoured to demonstrate the impossibility of any such violation of the second law. To name just a few paradigm-changing contributions, they include: Szilard's [11] analysis of the energetic cost of measuring molecular velocities, Brillouin's [12] use of the concept of negative entropy to link the concept of information to energy, Jaynes' [13] analysis of maximum thermodynamic entropy in informational terms and Landauer's [14] demonstration that the erasure of information (e.g. in computing) necessarily increases physical entropy, among many others.

These and many other related analyses showed that no physically implemented end-directed process can ultimately overcome the irreversibility and inevitability of the ultimate increase in entropy that is the consequence of the second law, at least not globally or in an isolated system. The assessment and selection processes that either Maxwell's demon or any corresponding mechanism would need to perform, itself involves physical work that increases entropy to an extent that more than counterbalances the entropy that it could reduce. Yet even though the actions of an intelligent agent cannot violate the second law, this does not close the door to the possibility of true teleological causality. It only describes what cannot be true of it.

## 3. Teleonomy versus natural selection versus cybernetics

Charles Darwin's theory of evolution by natural selection posed a roughly contemporary challenge to the concept of teleology. It is generally argued that the logic of natural selection offers a non-teleological explanation for the end-directed adaptations of living organisms. Adaptation evolves even though natural selection does not anticipate what sort of organism design will achieve a good fit with its environment. Any fit is the result of a *post hoc* consequence. But there is an often-unacknowledged thermodynamic issue that complicates this account. It has to do with a necessary ingredient of the natural selection algorithm: reproduction.

For natural selection to occur, multiple variant options must be produced and ‘tested’ with respect to one another. As the evolutionary biologist Maynard Smith [15] emphasized, this requires multiplication, not just reproduction. The available variant options must be generated well in excess of the number that will be able to persist. Some less successful variants need to be expendable. But these variant replicas do not just spontaneously appear, they must be produced. And reproduction requires thermodynamic work in the form of metabolism directed toward this end. So, although natural selection can explain away the appearance of design for a particular adaptive end, it nonetheless depends on metabolic and reproductive activities organized to counter the second law of thermodynamics locally.

In order to deal with the apparent end-directedness of organism physiology and reproduction, then, a mechanism that is more localized is necessary. Rosenblueth *et al.* [3] argued in ‘Behavior, purpose, and teleology’ that one could explain the apparent purposive behaviours of organisms in terms of negative feedback mechanisms. Classic examples of regulation by negative feedback are provided by the centrifugal governor of a steam engine or a thermostat controlling a household furnace. These devices are designed so that they initiate work to counter deviation away from some specified value or set point. The goal is to minimize variation away from the value of some physical parameter that is of importance for some function. The resulting behaviour is accurately described as end-directed toward this value.

This way of accounting for end-directedness in mechanistic terms motivated Pittendrigh [5] to coin the term ‘teleonomy’ as a neutral way of describing such dispositions in biology. Thus, he says: ‘The biologist's long-standing confusion would be more fully removed if all end-directed systems were described by some other term, like ‘teleonomic’, in order to emphasize that the recognition and description of end-directedness do not carry a commitment to Aristotelian teleology’. Describing the pattern of behaviour of a sea turtle this way one would say that it swims to the beach and lays its eggs rather than it swims to the beach in order to lay its eggs. Many years later, in response to Mayr [4], Pittendrigh wrote: ‘The crux of the problem lies of course in unconfounding the mechanism of evolutionary change and the physiological mechanism of the organism abstracted from the evolutionary time scale’. But can the process of evolution be abstracted away from the physiological mechanisms on which it depends without leaving its apparent end-directed distinctiveness unexplained?

Pittendrigh effectively acknowledges the primacy of organism metabolism when he wrote: ‘The most general of all biological “ends”, or “purposes” is of course perpetuation by reproduction. That end (and all its subsidiary “ends” of feeding, defense and survival generally) is in some sense effective in causing natural selection; in causing evolutionary change; but not in causing itself’ [4]. This leaves us with a chicken-and-egg dilemma. Is the thermodynamic work expended to maintain metabolism and facilitate reproduction a consequence of evolution? Is it an antecedent condition for natural selection to be at all possible? In abstracting the logic of evolutionary change from this ‘most general of all biological “ends”’ the thermodynamic distinctiveness of life is treated as orthogonal to the problem of end-directedness in nature. As a result, teleonomy neither explains teleology nor explains it away. It merely provides a way to avoid addressing the problem of teleology.

## 4. Thermodynamic implications

Teleological causal processes are defined by the target conditions they are presumed to bring about, but also by their non-spontaneous nature. Teleological causal processes have an ‘interventionist’ character, in this respect. This can only be explained if they tend to prevent an ongoing process or disrupt a stable condition. In thermodynamic terms, this orientation to the future can often be framed with respect to the second law, since the increase of entropy characterizes the ubiquitous spontaneous background context. So, commonly, teleological causal processes involve work that is produced to bring about conditions that would be thermodynamically unlikely to occur otherwise.

Consider such familiar human examples as using an umbrella to prevent rain from soaking one's clothes, wearing insulating clothes to prevent heat loss in cold weather, or eating to satisfy hunger. The first two cases involve ongoing physical processes that are thereby impeded. The example of eating also impedes a spontaneous process: the incessant energy expenditure that characterizes the metabolic work of staying alive. Because our organisms are maintained in a thermodynamically unstable far-from-equilibrium condition, degradation will spontaneously occur unless it is impeded by some energy barrier or compensated by work to counter this tendency.

This does not mean, however, that teleological causal processes are necessarily opposed to the increase of entropy. Many times, they boost it. For example, stirring coffee to increase the rate that sugar gets dissolved, or removing brush from a stream to unblock its flow, or chewing food to break up its structure and increase the surface area that will be exposed to digestive enzymes, all involve work produced to remove impediments to the increase of entropy and thereby increase its rate. Even though the intended result is to reach a state of increased entropy or equilibrium, this activity is end-directed because it involves work produced to counter a spontaneous tendency; in this case its rate of change, whether speeding it, impeding it or reversing it.

To summarize: each of these processes or activities exhibits two common features.

First, whether done consciously or produced automatically by an organism, each of these activities involves work produced to alter conditions that would otherwise persist, whether this is a spontaneous rate of change or an impediment to change.

Second—and less obvious from a thermodynamic perspective—in each case the work is produced in order to benefit some ultimate recipient, either directly or indirectly.

As we will argue below, both features can be understood in thermodynamic terms and ultimately with respect to opposing some background tendency that would transpire if unprevented. This background tendency might even be the product of some other contrary end-directed process, such as produced by another organism or challenging social relationship. But, in order to identify minimal thermodynamic determinants of teleological causality, the discussion that follows will focus on simple thermodynamic conditions.

## 5. Terminal versus target-directed processes

Colloquially, we describe teleological causal processes in terms of the ends that they produce (or are intended to produce). Thus, activity X is initiated ‘in order to’ bring condition Y into existence. Y is the end for which X was produced, and presumably Y is less likely to come into existence without X increasing this probability. This is our common sense understanding of purposeful action. But ends in the form of states toward which changes tend are ubiquitous. So, what distinguishes teleological causal processes from other self-terminating processes?

Many natural processes that we would not consider to be teleological contribute to asymmetric changes toward specific end states. For example, objects thrown into the air are pulled toward the centre of the earth by the force of gravity and fall back to earth until they come to rest on the ground; opposite poles of two magnets will pull each other closer until they touch; sugar mixed into tea will dissolve and disperse evenly until the concentration is everywhere the same; and frozen food left out to thaw will eventually reach a temperature that is at equilibrium with the surroundings.

We will call the processes that are the simple result of global asymmetries ‘terminal’ because they involve spontaneous change toward a terminal (either maximum or minimum) value of some variable beyond which no further asymmetric change can occur. Aristotle saw nature as pervaded with lower-level terminal tendencies. In his view of the cosmos, he believed that basic elements like earth, air, fire and water spontaneously tend to move toward their ‘natural levels’ unless this is prevented by external forces.

In this respect, the second law of thermodynamics describes a terminal process. Unless impeded from changing state, a physical system of many dynamically interacting parts will spontaneously increase in entropy until it reaches its maximum entropy value, at equilibrium, where asymmetric processes of change are in balance. External boundary conditions determine the maximum and minimum values that some state variables can assume. When this value is reached asymmetric change ceases. In this respect, the maximum entropy state of a thermodynamic system may be characterized as its terminal state; i.e. the point where further change in that direction is impossible. Given that a relentless spontaneous increase of entropy accompanies physical transformations of all kinds, the second law is the indirect source of many other asymmetric changes that are irreversible and with respect to which teleological process are often distinguished.

Note that, because terminal states, while superficially end-directed, lack any satisfaction conditions, they do not ‘attract’ change in that direction, nor do they ‘force’ things toward a terminal value. No work is being performed to pull or push things toward a terminal end-state. These end-states are, instead, a reflection of global asymmetries in the possible trajectories of change constraining local causal processes. In the case of gravitational interactions, it is the mass and distance that matters. In the case of a chemical reaction, it is relative concentrations, ambient temperature, molecular valences, etc. that matter. This has been likened to a field effect [16] in which any directionality to change is determined extrinsically, by what might be described as boundary conditions that determine which attributes of a thing are involved in any transformation. As a result, the terminal point at which a change of state ceases is a function of these external relations.

Interestingly, Babcock and McShea argue that teleology is a localized expression of extrinsically converging field effects. This leads them to claim that there can be no intrinsic teleological property. We agree that all causal dispositions are the result of the convergence of extrinsic influences, and that to attribute teleological processes to the constituents of an organism risks introducing an ineffable teleological property that merely begs the question of its causal basis. By contrast, however, we will demonstrate how teleological processes in biology can arise from the organization of local dynamical processes that reciprocally produce each other's boundary conditions. As a result, critical field effects external to each component process are produced intrinsic to the whole and can thereby counter external background field effects. In this sense, the theory of biological teleology we propose is both consistent with strict externalism but neither reducible to the convergence of external relations nor attributed to any sort of ‘elan vital’. For a more detailed critique of the field theory and its denial of teleological internalism, see García-Valdecasas [17].

By contrast to terminal processes, the processes that we will call ‘targeted’ or ‘target-directed’ only ‘terminate’ with respect to internal boundary conditions. These conditions embody the values of some localized system-internal parameter. As a result, these processes are neither spontaneous nor distributed. They involve work that appears to be initiated from localized sources to prevent specific outcomes. And they produce patterns of change that deviate from what external boundary conditions would otherwise impose.

For example, lifting an object off the floor to set it on a higher surface, pulling apart magnets held together by magnetism, putting on a coat to reduce the rate at which body heat dissipates, are all activities that run counter to a spontaneous terminal tendency implicit within the larger context in which these activities occur. Even if the resulting change is parallel to thermodynamic change, such as stirring tea to speed up the rate that sugar dissolves, it nevertheless modifies what would tend to occur spontaneously. The ends of such end-directed actions are specific nonterminal states. They are ‘target’ states that are not where things would tend to settle were it not for these interventions whose source has a local origin.

‘Targeted’ or ‘target-directed’ processes are genuinely teleological. Still, the challenge is to demonstrate how critical boundary conditions can become internalized so that they can at least temporarily overcome the influence of external boundary conditions.

## 6. From terminal to target-directed

To characterize a given dynamical transformation it is useful to identify which state parameters of the system remain invariant, which state parameters change, and the lower and upper bounds of this capacity to change. In classical thermodynamics, the first two of these loosely correspond to the first and second laws of thermodynamics.

Absolute zero temperature, which can be approached asymptotically, is generally recognized as the ultimate lower bound for any physical system, and in any particular transformation, there is also a point at which the entropy of the system cannot increase any further without a change in its constituents. In an isolated system, this is its maximum entropy state—its state of equilibrium. This too tends to be approached asymptotically such that fluctuations below this value are possible, but beyond which an increase in entropy is no longer possible.

In addition to these most basic principles for characterizing any dynamical system, thermodynamics is likewise characterized by the asymmetry of the second law. The state parameter that changes in any physical transformation is its entropy, and at least in a system isolated from outside influence, its entropy can only increase, and its free energy or capacity to do work can only decrease. This further determines that work is required to reduce the entropy of a system while an increase of its entropy occurs spontaneously. As a result, living processes must perform thermodynamic work to maintain themselves far-from-equilibrium internally, and in so doing, export entropy into the surroundings. And they must be able to continue to do this despite the boundary conditions of the surrounding environment. In other words, they must find a way to internalize their own boundary conditions.

To see how this might be possible we need to first consider the distinctive terminal dynamics of far-from-equilibrium dissipative processes. Such dissipative ‘structures’ as vortices in air or water and the hexagonal tessellation of Rayleigh–Bénard convection cells in a heated oil are often described as self-organizing because their highly regular geometry is not imposed extrinsically. Only constant perturbation or extreme displacement away from equilibrium is imposed. The farther a system is displaced from equilibrium the more ‘free energy’ is directed to reaching this end state. Regularity develops under these conditions as dissipation is channelled more and more directly, offloaded entropy into the surrounding more effectively.

Over and above the rate that an energy gradient would dissipate spontaneously, this accelerated dissipation occurs because less regular dissipative processes become overrun by more efficient, more regular ones. In the process, dissipation is maximized within the constraints of the medium. This tendency is sometimes referred to as the maximum entropy production principle (MEPP) (see e.g. [18]).

In recent decades, the study of self-organizing dissipative processes has become a major focus with respect to the thermodynamics of life. Its importance was hinted at by the physicist Erwin Schrödinger in his 1944 book *What is Life* that explored the distinctive characteristics of life from a physicist's point of view. In this work, he mused that the tendency for organism entropy to increase needed to be compensated by acquiring what he called ‘negentropy’. But it was the pioneering work of thermodynamic theorists such as Prigogine [19] and Eigen [20], exploring the dynamics of processes far-from-equilibrium, that brought attention to the special character of self-organization.

Because living systems are far-from-equilibrium low-entropy phenomena they are intrinsically precarious. The possibility of persistence, therefore, requires a constant source of thermodynamic work to maintain this intrinsically unstable state including maintaining any constraints preventing its degradation.

This explains why self-organizing processes are critical to living processes. Superficially, an obvious relevance of self-organizing dynamics to life is that such processes tend to locally increase order and decrease local entropy. This has led to considerable interest in the possibility that life can be described as just a complex self-organizing system (e.g. see [18,21,22]). Although self-organizing processes require a constant source of externally provided energy and material resources and must offload entropy into the environment, this is what enables them to reduce local entropy. This generates local constraints on energy and material flow in the form of physical structures and dynamical regularities. These, in turn, are what channel energy utilization to continue to do this work.

But there are at least three critical problems with the life = self-organization equation.

First, self-organizing processes are intrinsically self-undermining. Their increased organization (local entropy reduction) is a consequence of maximizing the rate that entropy is passed through. This means that they maximize it at the same time that they reduce the very energy gradient that creates these regularities. This is not a problem so long as this gradient is available, but it significantly limits the conditions in which they can be sustained.

Second, the particular dynamical regularities they exhibit are entirely a consequence of the gradient that drives them and their material constituents. Although the term ‘self’ in self-organization refers to the fact that the particular forms produced are not imposed from the outside, neither are they imposed internally. They result from the interaction of internal material constraints and the form of external perturbation. There is no individual ‘self’ that is the source of this effect.

Third, because this form is not embodied by any intrinsic feature, it cannot replicate. It can only propagate by perturbing the dynamics of adjacent regions (as demonstrated by Rayleigh–Bénard convection cells or vortex streets).

These three properties make self-organized processes merely terminal processes. The stable regularities that result—though transiently in the case of depletable energy gradients—are a function of reaching a maximum level of possible entropy generation. And if this eventually depletes the gradient that generates this regularity, so that they eventually degrade toward a maximum entropy terminal state near equilibrium.

This leaves us with a conundrum. In order to generate and maintain organization, living processes must take advantage of self-organizing processes, and yet they must also prevent these processes from depleting the very gradients that drive them. So, how can life both use self-organization at the same time that it prevents or holds off its terminal tendencies?

## 7. Teleodynamics

This conundrum can only be resolved by recognizing that different self-organizing dynamics, driven by independent gradients and subject to different dynamical properties, interact in ways that complement one another. To see how this synergistic relationship may be possible it is useful to consider a simple model system. This model has been developed by Deacon [23,–26], to explore issues involving the origin of life and the nature of genetic information as well as the nature of end-directed processes. Here, we will only use it to explore how self-organized processes, which are by themselves terminal processes, might have become combined to give rise to targeted dynamics.

Beginning with this simple model system is a necessary first step, because the thermodynamical organization of even the simplest autonomously living cellular organisms—bacteria—is incredibly complex. The tools needed to disentangle the elaborate webs that constitute physical–chemical interactions of such systems are currently unavailable. But even if they were, it would be difficult to disentangle the minimally essential dynamics from the immense web of interlinked dynamical processes that have evolved in even the simplest of cells over the course of nearly 4 Gyr.

So, we need a model that is as simple as possible, but not so simple that the properties of interest are missing. What needs to be included in such a model? The thermodynamic riddle that needs to be answered in order to provide a physically grounded notion of teleological causality is this: How can the interactions among different terminal processes be organized so that they collectively converge to a target state that both diverges from any component terminal state, and yet also preserves their inherent dispositions to contribute to their collective preservation?

A key feature of the self-organizing component processes of organisms that makes this possible is that entropy reduction is also a reduction in the degrees of freedom of possible change; i.e. increased constraint. Physical constraints can be embodied in many different forms, from dynamical regularities to physical barriers to distribution biases. Moreover, the boundary conditions that can support or inhibit a particular form of self-organizing dynamics are environmental constraints on the energy and material flows that support it. Consequently, it should be possible to identify distinct self-organizing processes that each reciprocally generate each other's permissive boundary conditions, and collectively prevent each other from reaching terminality at equilibrium.

By impeding each component self-organizing process from reaching its terminal state, such a system will be disposed to develop toward a target state that preserves the terminal dispositions of each process but also prevents them from reaching termination. Such a system of linked self-organized processes will tend to preserve the local constraints that each process generates before the whole system depletes its resource base. In other words, the system will develop toward a target state that is not a terminal state. But when perturbed away from this target state the collective actions of the component self-organizing processes will tend to return the system to that state again. This might be described as a basic form of self-repair.

A molecular 'model system' that exhibits these properties is described below. It is effectively an ‘autogenic virus’ (‘autogen’ for short). It is modelled on simple viral molecular architecture but is ‘autogeneic’ because it can repair and reproduce itself autonomously. Although an autogen could be superficially described as a version of an autopoietic system [27], it is its thermodynamic organization, not its behavioural properties—such as autonomy or constraint closure [28]—that defines it. Analysing its hypothetically likely dynamic properties can help to illuminate the thermodynamic requirements of teleological causality.

The basic thermodynamic characteristics of autogenic dynamics and its relationship to near- and far-from-equilibrium thermodynamical processes was formally described in a presentation and paper produced for the 2020 Thermodynamics 2.0 conference (see [25]). And a more detailed account exploring the relevance of this model to biological information can be found in Deacon [26]. Given this, it will only be briefly described below.

Consider a simplified description of viral biology. A simple virus is a structure involving two distinct kinds of molecular components: a protein shell (called a capsid) that contains a nucleic acid polymer like RNA or DNA. If it infects a host cell, the capsid breaks open releasing its nucleic acid contents. The cell mistakes it for its own and begins to both make copies of it and translate its nucleotide code into capsid protein molecules. Within the cell the new capsid molecules self-assemble (roughly analogous to crystal formation) into new capsid containers that contain the newly replicated viral nucleic acid polymers. If eventually, the cell bursts open the many new viral replicas are released to infect other cells. Outside of a cell, they are relatively inert molecular structures that can often persist in diverse and somewhat non-supportive conditions.

An autogenic virus, in contrast, is not parasitic in this way. Lacking the complex DNA–RNA replication and translation machinery of a cell, its contents need to be able to contribute to their own replication and the production of capsid molecules more simply and directly. A process that can accomplish both features is reciprocal catalysis—also called a collectively auto catalytic set of molecules (see [21]). In its simplest form, two catalyst molecules A and B each catalyse the formation of each other via catalysing substrate molecules. Since the catalysts are not themselves transformed in the process, this will result in an accelerating increase in catalyst concentrations in a local region, as well as a decrease in substrates and the production of side products to the point at which catalysis formation reaches a maximum. When this is reached, its rate no longer outpaces the diffusion of reciprocal catalysts and their probability of interacting decreases. In other words, this is a self-organizing terminal process. But this terminal state can be postponed if, before substrates are used up, the reciprocal catalysts are encapsulated together so that they can neither diffuse away from one another nor use up substrates. This is possible if, like viruses, these molecules become encapsulated within an impermeable capsid shell.

Capsid shell formation—like the growth of a crystal lattice—depends on the capacity of both the reciprocal shape and properties of capsid molecules that promotes them to fit regularly together into a tessellation, and also their relative concentrations that affect the rate at which they tend to attach or detach from a growing lattice. As the capsid shell grows, it will progressively reduce capsid molecule concentration in the surrounding solution until it reaches an equilibrium point where capsid molecule attachment and detachment from the lattice are balanced. This is the terminal state of the crystalline self-assembly process. Another possible terminal state for capsid growth is also characteristic of most viral capsids: growth into a finite closed polyhedral container to which additional molecules cannot be added. Even if the molecular geometry of the capsid molecules predisposes polyhedron formation, this end can only be achieved if capsid concentration remains high in the surroundings despite being reduced by capsid growth.

**Figure 1.**
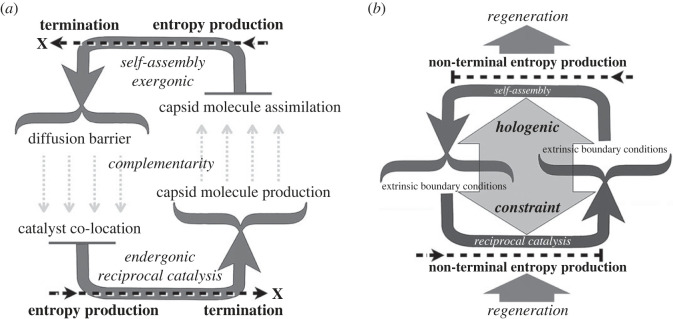
This diagram depicts how linked interdependent self-organizing processes can give rise to intrinsically determined target-directed causality. (*a*) Two distinct far-from-equilibrium self-organizing processes—self-assembly (top) and reciprocal catalysis (bottom)—are depicted as large dark grey arrows along with the exergonic (spontaneous) or endergonic (non-spontaneous, energy driven) character of these processes. Their complementary products and boundary conditions are labelled and their complementary relationships to one another are indicated by dashed light grey arrows. The terminal entropy-producing character of each self-organizing process is indicated by dashed black arrows. (*b*) When physically linked by a shared capsid molecule these self-organizing processes become yoked into a higher-order synergistic thermodynamic unit. This facilitates the persistence of both self-organizing processes, prevents their separate development to irreversible termination, and internalizes the potential to preserve the organizational integrity of the whole. This higher-order constraint on the co-dependence of these component constraint-producing processes creates a self-preserving individual with distinctive thermodynamic properties. It is described as hologenic because it constitutes the indecomposable basis for the self-perpetuating thermodynamic unity of the autogenic virus.

These two processes are common in the living world. Comparing them, an interesting complementarity can be discerned. If one of the side products produced by reciprocal catalysis is a capsid-forming molecule, then increasing the production of catalysts will also continually increase capsid molecule concentration. This increase could compensate for the decrease caused by capsid growth. In this case, capsid shell formation would tend to occur most effectively in regions undergoing the most rapid reciprocal catalysis. This, in turn, would increase the probability of capsid shells enclosing the catalysts and both stopping catalysis by separating them from substrates and preventing them from diffusing away from one another. This relationship is diagrammed in figure 1.

In other words, each of these self-organizing terminal processes—reciprocal catalysis and capsid shell self-assembly—generates the boundary conditions that the other requires, but in addition prevents the other from reaching an irreversible terminal state. As a result, the synergistic coupling of both processes will develop toward a target state that, although relatively inert, preserves the potential for both self-organizing capacities to recur when conditions are right.

This targeted disposition is teleological (i.e. future-oriented). This can be illustrated by considering the self-reparatory and self-reproductive character of such a system. If the capsid shell of an autogenic virus is somehow breached or disrupted in the context of sufficient substrates and conditions, both reciprocal catalysis and capsid self-assembly will recommence. The process will continue until the complex becomes closed, inert and relatively stable—like a virus particle outside of a cell. And this self-repair process could even result in the formation of more than one autogen if excess components become more widely dispersed. In this respect, the target state embodies the potential to both ‘remember’ this intrinsic ‘self-description’ and to potentially transfer it to new molecular complexes. In effect, the intrinsic target-directed disposition of the autogen embodies the potential to project itself into the future. In this way, even such a minimal teleologically organized system is evolvable. Because it can transfer its causal organization to new substrates, a trace of prior adaptations is preserved, with respect to which new variants can be added and ever more complex forms of self-repair can evolve.

## 8. Conclusion

An autogenic process is produced by the synergistic coupling of two or more terminal processes. These terminal processes must be far-from-equilibrium self-organizing processes that each produces the supportive and limiting boundary constraints for the other. As a result, the local entropy-decreasing dynamic of each is facilitated while their tendency to develop self-termination is prevented. The co-dependent coupling of these self-organizing dynamics thereby creates a higher-order dynamical synergy with a disposition to repair damage to its integrity and to reconstitute its individual unity if damaged, despite radical material replacement. The model we describe demonstrates that at least two co-dependently coupled self-organizing processes are both necessary and minimally sufficient to produce a locus of teleological causality. More complex teleological organizations are possible, and are likely to evolve once minimal teleological agency emerges.

This recursively co-dependent relationship between constraint-producing processes could potentially be embodied in a range of molecular substrates. In this respect, it is multiply realizable. So, although we have described a minimal teleological system in terms of known molecular processes, there are potentially a great many physical processes that could be organized autogenically. This multiple realizability demonstrates that maintaining the continuity of these unifying constraints is its target state; a state which is capable of being imposed on new substrates in future conditions. The result is an organized system that acts on its own behalf to preserve its existence when its unity is at risk, and even potentially reproduces itself in conditions of sufficient surplus and dispersion.

The specific molecular model of autogenesis described here as an autogenic virus [26] is not merely a thought experiment, but a potentially realizable entity constituted by molecular processes that are commonly found in living systems. This makes this theoretical claim empirically testable. Though vastly simpler and lacking any semblance of subjectivity, the basic form of this molecularly based causal potential warrants describing it as teleological rather than merely teleonomic. This is because it not only exhibits end-directed behaviour, but also because the target of this behaviour is of intrinsic value to the system that produces it. In this respect, the process we have called autogenesis constitutes a minimal form of teleology that exists for its own sake.

Analogous to the way a mental representation of a potential future state contributes to purposive action, this minimal teleological disposition re-presents the abstract form of a future state and influences the probability that this form will be realized in some not-yet-existing physical condition. So, although autogenesis is vastly simpler than mental purpose, it nevertheless provides a naturalized model of teleological causation that both escapes the threat of backward influences and does not reduce teleology to the effects of selection, feedback or mere description. Understanding the thermodynamic basis of this most minimal form of teleological dynamics is a necessary first step toward explaining the emergence of mental agency.

## Data Availability

This article has no additional data.
